# Successful Treatment of Exploding Head Syndrome With Ketamine

**DOI:** 10.7759/cureus.97558

**Published:** 2025-11-23

**Authors:** Luiza Harutyunyan, Mitchell Liester, Bertrand Liang

**Affiliations:** 1 Department of Neurology, St. Joseph's Medical Center, Stockton, USA; 2 Department of Psychiatry, University of Colorado Anschutz Medical Campus, Denver, USA; 3 Department of Neurology, University of Colorado Anschutz Medical Campus, Denver, USA

**Keywords:** exploding head syndrome, ketamine, parasomnia, sleep, treatment

## Abstract

Exploding head syndrome (EHS) is a parasomnia characterized by the perception of loud noises originating from inside the head during sleep transitions, often accompanied by visual phenomena and fear. Treatment remains challenging due to unknown etiology and limited therapeutic options. We report a 75-year-old man with chronic exploding head syndrome experiencing lightning-like sensations, thunder-like sounds, sleep paralysis, and intense fear during sleep onset. Episodes occurred multiple times weekly for over five years. Initial treatments, including gabapentin, valproic acid, amitriptyline, and buspirone, proved ineffective. The patient was subsequently treated with sublingual ketamine, starting at 25 mg every third night and escalating to nightly dosing. After one month, episode frequency decreased from 3-4 times weekly to once every two weeks. By three months, episodes occurred monthly with reduced intensity. After six months, the patient experienced only occasional sleep paralysis with the complete resolution of exploding head syndrome and reported improved quality of life. Ketamine's mechanism likely involves N-methyl-D-aspartate (NMDA) receptor modulation, brain-derived neurotrophic factor release, and σ1 receptor agonism, promoting neuroplasticity and sleep regulation. This case represents a reported successful treatment of exploding head syndrome with ketamine, suggesting a potential therapeutic approach for this refractory parasomnia. Further studies are warranted to evaluate ketamine's efficacy in exploding head syndrome treatment.

## Introduction

Sleep disorders are unintentional physical events that occur at the beginning of sleep, while sleeping, or when aroused from sleep. Parasomnias are sleep disorders where patients are partially aroused from sleep, such as sleepwalking, sleep terrors, and sleep paralysis. The brain cycles through multiple states during sleep, including wakefulness, nonrapid eye movement (NREM) sleep, and rapid eye movement (REM). NREM sleep is divided into three stages, including stage I (transition between awake and sleep), stage II (the majority of the sleep period), and stage III (slow-wave sleep). Parasomnias commonly occur between stage III/slow-wave sleep and wakefulness, leading to behaviors without full awareness and mentation related to the awake state, although some parasomnias occur in both NREM and REM sleep [1]. In particular, exploding head syndrome (EHS) is a parasomnia involving both REM and NREM sleep. The prevalence of recurrent EHS has been estimated at between 4% and 7% [1,2]. Patients describe the phenomenon as a loud and sudden noise developing from inside the head; noises have been variably described, including gunshots, explosions, lightning, and fireworks [2]. In addition to hearing these abnormal noises, patients may also report visual phenomena, such as flashing lights, during these events. Associated headaches are not reported, but patients express fearfulness during these events [2]. Exploding head syndrome has been found to be difficult to treat, with various approaches including sleep hygiene, cognitive behavioral therapy, and pharmacologic interventions being of only modest effectiveness [3]. There are no approved agents for EHS. The differential diagnosis includes primary headache syndromes, post-traumatic stress disorders, panic attacks, and seizures [1,2]. This case describes the rare occurrence of exploding head syndrome associated with sleep paralysis and lightning sensations successfully treated with ketamine.

## Case presentation

A 75-year-old man with a history of hypertension and diabetes presented to his primary family practice physician with vague complaints about pain. He had initially been treated with nonsteroidal anti-inflammatory agents (ibuprofen) without effect and subsequently within the neurology department with anti-migraine agents (sumatriptan, propranolol, and amitriptyline) and muscle relaxants (cyclobenzaprine) unsuccessfully. The patient was seen by a psychiatrist, who diagnosed the patient with mild anxiety without placing the patient on medications. The patient was then referred to the sleep disorder clinic, as one clinician noted the patient's pain had been associated with sleep.

The patient described the pain as "lightning-like," emanating from his head and extending to the rest of his body. He would experience a "flash" in his head and then a feeling of shock in his body with a loud "bang" (described as "thunder"). These pains had been chronic, occurring several times per week for over five years, without known exacerbating factors. The patient reported that these events typically occurred while falling asleep, although they occasionally occurred during sleep, resulting in awakening. Sleep paralysis accompanied the episodes. In each event, there was a feeling of "terror" as described by the patient, and overall, each event lasted about 10 seconds. Otherwise, the patient did not describe any difficulty falling asleep (although he noted at times fearing going to sleep due to the lightning-like experience), any snoring, or excessive daytime sleepiness. A review of systems was negative except for using an e-reader at night before going to bed for a "calming effect." There was no history of epilepsy nor hypnogogic hallucinations.

The patient noted that there were no major stressors in his life; he had been compliant with medication for his hypertension (losartan) and diabetes (metformin), having been taking these medications for over a decade. He was taking no other medications. The patient noted that he ate a ketogenic diet; he denied alcohol, tobacco, or illicit drug use. Family history was significant for non-Hodgkin lymphoma in the father and a myocardial infarction in the mother.

The patient's Epworth sleep score was noted to be 1/24 with a Mallampati score of II. BMI was 20.1. Vital signs included a respiratory rate of 16, blood pressure sitting of 122/79, pulse of 68 beats per minute, and temperature of 37.2 degrees Celsius. Neurologic examination revealed no abnormalities except a slight flattening of the left nasolabial fold.

The patient's laboratory tests revealed normal iron, ferritin, vitamin B12, complete blood count, and basic metabolic panel; an MRI scan showed evidence of mild microvascular disease but was otherwise normal without infarct, edema, or mass effect; EEG was unremarkable.

The patient underwent a sleep study (Table 1). Total sleep time was five hours and 22 minutes. Sleep latency was 14 minutes with a sleep efficiency of 88%. The primary sleep stage (the stage spent the most time in sleep) was stage II (3.7 hours), with 61 minutes in REM sleep. There was no evidence of obstructive or central sleep apnea noted nor evidence of (other) parasomnias. The patient did not experience a lightning-like episode during the sleep study.

**Table 1:** Sleep study REM, rapid eye movement; bpm, beats per minute

Parameter	Value	Normal range
Total sleep time	5 hours and 22 minutes	>360 minutes
Sleep latency	14 minutes	10-20 minutes
Sleep efficiency	88%	85%-95%
Primary sleep stage	Stage II (3.7 hours)	3.5-4.5 hours
REM sleep	61 minutes	60-120 minutes
Apnea-hypopnea index	1 event total	<5 per hour
Minimum SpO_2_	91%	>90
Periodic movement disorder index	1 event total	<5 per hour
Mean heart rate	62	50-80 bpm during sleep

The impression was that the patient had a parasomnia, the exploding head syndrome (EHS). The patient was initially begun on gabapentin for a month, which did not relieve the symptoms; he was then tried on valproic acid (500 mg three times a day {tid}) and amitriptyline (up to 50 mg every day {qd}) for a month each, which were also ineffective. The patient was begun on buspirone, which over the ensuing month decreased the intensity of the thunder sound but not the frequency. He discontinued the medication after nine months, noting no additional improvement. The patient was then begun on sublingual ketamine, with dose escalation from 25 mg every third night to nightly over three weeks. After one month, the patient noted improvement with a decreased frequency from three to four times a week to once every couple of weeks. After three months, the patient was having only one episode per month, which was diminished in intensity. After six months of treatment, the patient noted only occasional sleep paralysis, no EHS, and an "improved quality of life." The patient subsequently moved from the United States back to his native country and was no longer available for follow-up.

## Discussion

This case demonstrates the successful use of ketamine for the treatment of EHS. Ketamine was originally known as an anesthetic, synthesized in the 1960s, and approved for use as a general anesthetic in 1970. Ketamine at high doses exerts neurotoxic effects through presumed excitotoxicity. In contrast, at lower doses, it demonstrates neuroprotective effects, which are likely related to the release of the neurotrophin brain-derived neurotrophic factor (BDNF) [4]. There have been cases of low-dose sublingual ketamine improving treatment-resistant depression, as well as bipolar disorder [4]; indeed, the low dose of 0.5 mg/kg of IV ketamine has been shown to provide significant improvement in depression in a 72-hour interval. However, a lower dose of 10 mg given sublingually and repeated every two to seven days exhibited quick and sustained improvement in cognition, mood, and sleep in patients with treatment-resistant depression [4]. A study comparing esketamine effects in patients with and without insomnia found that after six months, patients with insomnia showed significant improvements in insomnia severity (p = 0.016) and next-day well-being (p = 0.003), reaching levels comparable to patients without baseline insomnia [5].

Ketamine's mechanism of action involves (at least) modulating N-methyl-D-aspartate (NMDA) receptors and α-amino-3-hydroxy-5-methyl-4-isoxazolepropionic acid (AMPA) receptors [4]. This modulation induces neuroplasticity and stabilizes the neurotransmitters needed for regulating sleep. It has thus been postulated that ketamine and ketamine derivatives affect NMDA receptors, which in turn affect the sleep cycle and reduce the time needed to fall asleep and reduce awakenings at night [5].

Ketamine has also demonstrated a role in Wolfram syndrome. *WFS1* mutations lead to reduced import in Ca 2+, causing mitochondrial dysfunction, which has been linked to depression. Ketamine acts as a σ1 receptor agonist, improving the psychiatric symptoms associated with this syndrome. Specifically, R-ketamine has been shown to have a strong affinity for σ1 receptors. This was shown in a drug-discrimination study where a σ1 receptor antagonist blocked the drug cue induced by R-ketamine. Additionally, preclinical depression models demonstrated that σ1 receptor antagonists blocked ketamine's antidepressant effects [6].

Several psychedelic medicines are also known to enhance neuroplasticity by promoting neurogenesis through interactions with σ1 receptors similar to the R-enantiomer of ketamine. At low doses, these substances have also been shown to improve behavioral responses related to stress and depression [7]. Specifically, N,N-dimethyltryptamine (DMT), a psychoactive alkaloid in the South American plant medicine ayahuasca, provides neurogenic effects. Mice models have shown that σ1 receptor activation is involved in this neurogenic effect, which leads to the downstream effects of improving adult neurogenesis and enhancing memory tasks and spatial learning [8].

A structurally similar compound, 5-methoxy-N,N-dimethyltryptamine (5-MeO-DMT), has also demonstrated therapeutic efficacy for mood disorders. A murine study demonstrated that those treated with 5-MeO-DMT had an increased number of dentate granule cells. The dentate gyrus is an area where neurogenesis is maintained through adulthood [9]; this provides further evidence of potential actions of these compounds within the nervous system and targets by which parasomnias may have involvement.

The use of ketamine for the treatment of EHS has not been previously reported. Although this syndrome has been related to emotional stress and poor sleep quality, the underlying etiology is unknown, and our patient did not report either clinical aspect [10]. EHS has also been known to be refractory to treatment, which may be attributed to the unknown pathophysiology of the syndrome. Indeed, there have not yet been clinical trials on the treatment of EHS. Many patients can be reassured about the benign nature of the condition, and in some cases, drug therapy is not necessary [11]. Although there is no direct evidence demonstrating a pathophysiological role of NMDA receptors, BDNF, and σ1 receptors in EHS, it is speculated that there could be a downstream effect leading to neuroplasticity and the stabilization of neurotransmitters and potentially improving parameters of the sleep cycle and thus parasomnia symptomatology. Further correlative studies will be required in order to assess this hypothesis. Nonetheless, in refractory patients, the use of low-dose ketamine may be an option for those suffering from EHS.

## Conclusions

This case report demonstrates the rare phenomenon of EHS successfully treated with low-dose ketamine. While the effectiveness of ketamine cannot be directly established in EHS, the results are intriguing nonetheless. Although the patient had tried anti-epileptic medications, a tricyclic antidepressant, and an anxiolytic, they were all ineffective in improving syndrome symptoms. Although there has not been direct molecular data on the effects of ketamine on this syndrome, there may be downstream pathophysiological effects related to the NMDA receptor, BDNF, and σ1 receptors, which may lead to the neuroprotective, neuroplastic, and neurogenic mechanisms that improve sleep regulation. Further evaluation of the use of ketamine in EHS and the assessment of its potential use in parasomnias may provide a promising approach for future patient care.
